# Universal Rapid Human Immunodeficiency Virus Screening at Delivery: A Cost-Effectiveness Analysis

**DOI:** 10.1155/2018/6024698

**Published:** 2018-03-14

**Authors:** Rachel K. Scott, Stacia Crochet, Chun-Chih Huang

**Affiliations:** ^1^MedStar Health Research Institute (MHRI), Washington, DC, USA; ^2^MedStar Washington Hospital Center (MWHC), Division of Women's and Infants' Services, Washington, DC, USA; ^3^Emory University Hospital, Atlanta, GA, USA; ^4^Georgetown-Howard Universities Center for Clinical and Translational Science, Washington, DC, USA

## Abstract

**Objective:**

To determine the cost-effectiveness of universal maternal HIV screening at time of delivery to decrease mother-to-child transmission (MTCT), by comparing the cost and quality-adjusted life years (QALYs) of universal rapid HIV screening at time of delivery to two current standards of care for prenatal HIV screening in the United States.

**Study Design:**

We conducted a cost-effectiveness analysis to compare the cost and QALY of universal intrapartum rapid HIV screening with two current standards of care: (I) opt-out rapid HIV testing limited to patients without previous third-trimester screening and (II) opt-out rapid HIV testing limited to patients without any prenatal screening. We developed a decision-tree model and performed sensitivity analyses to estimate the impact of variances in QALY, estimated lifetime medical costs, HIV prevalence, and cumulative incidence.

**Results:**

The incremental cost-effectiveness ratio for universal screening was $7,973.45/QALY. The results remained robust to sensitivity analysis, except for annual cumulative incidence. In areas with an annual cumulative incidence rate of <0.02% for reproductive-age women, the incremental cost-effectiveness ratio for the expanded program would exceed $89,926.94/QALY, approaching the commonly applied cost-effectiveness thresholds ($100,000/QALY).

**Conclusions:**

Intrapartum universal rapid HIV screening to decrease MTCT appears cost-effective in populations with high HIV incidence in the United States.

## 1. Introduction

Mother-to-child transmission (MTCT) of Human Immunodeficiency Virus (HIV) is largely preventable through early diagnosis and antiretroviral therapy (ART), including maternal and neonatal prophylaxis globally and cesarean delivery (CD) for high or unknown viral load in resource-rich settings. The risk of MTCT is significantly elevated among pregnancies with incident HIV infection compared to pregnancies with known maternal HIV [1]. In more than a quarter of cases of MTCT in the United States, maternal HIV infection is not diagnosed until after delivery [2, 3], obviating or delaying measures to prevent transmission. The Centers for Disease Control (CDC) and the American College of Obstetricians and Gynecologists (ACOG) both recommend HIV screening as early as possible in pregnancy and rapid screening in labor if patients have not had prior prenatal screening. In high risk patients, high incidence and prevalence communities, and medical centers with a screen-positive rate of greater than one per 1,000, ACOG recommends additional third-trimester screening prior to 36 weeks of gestation [2–5]. Based upon the CDC and ACOG recommendations, the standard of care in high prevalence communities is to perform a rapid HIV screen on patients in labor who have not had third-trimester screening and in low-incidence and low risk populations is to perform rapid screening in labor if there has been no previous HIV screening during pregnancy.

Most children born with perinatally acquired HIV (PAH) now survive to adulthood; however, not without both medical and behavioral health consequences [6–8]. In addition to the long-term effects of HIV infection, the adolescents and young adults who have grown up with PAH frequently have virologic resistance from inconsistent long-term ART use. Additionally, this patient population has demonstrated increased high risk sexual behavior, mental illness, and substance abuse [7, 9, 10]. From birth, these individuals face a lifetime of chronic disease, as well as the associated stigma and resulting behavioral consequences and health comorbidities.

We hypothesized that* universal*, opt-out, rapid HIV screening upon admission for delivery (either vaginal or CD) to Labor and Delivery (L&D) provides an additional opportunity, in high incidence and prevalence populations, to prevent MTCT in mothers who were not previously diagnosed with HIV, secondary to either lack of prenatal screening or seroconversion in the third trimester of pregnancy. Diagnosis and treatment of intrapartum HIV cannot prevent MTCT that may have already occurred in utero; however maternal intravenous zidovudine (AZT) prophylaxis, CD before active labor or rupture of membranes (ROM), and enhanced neonatal antiretroviral prophylaxis with 2 or 3 ART all independently decrease the risk of intrapartum MTCT [11–14]. Recognizing the logistical challenge of intrapartum AZT prophylaxis due to the time limitation of active labor and the unclear benefit of CD during active labor or after ROM, at minimum, the diagnosis of HIV in labor allows for immediate neonatal prophylaxis postpartum, which alone has been demonstrated to decrease perinatal transmission to 2.2–2.4% [12] (from 25.5% without intervention [15]), the opportunity to counsel against breastfeeding, and early diagnosis and treatment of neonatal HIV.

The prevalence of HIV among women in the District of Columbia (DC) is over eightfold higher than the national average (1.4% compared to 0.17% nationally) [16]. The DC Department of Health estimates the prevalence to be even higher among reproductive aged women. Given the high prevalence (1.9%) and annual incidence (0.087%) among reproductive-age women [17], our medical center recently implemented universal rapid HIV screening of all patients with viable pregnancies admitted for delivery. This study evaluated the cost-effectiveness of universal rapid HIV screening at time of delivery in a high HIV prevalence and incidence area, such as DC.

## 2. Methods

### 2.1. Study Model

We set out to test our hypothesis that universal rapid HIV screening would be cost-effective in high incidence and prevalence areas of the United States, secondary to identification of previously undiagnosed maternal HIV and resulting in timely intrapartum and postpartum interventions to prevent MTCT and improve neonatal outcomes. We conducted a cost-effectiveness analysis from the societal perspective to compare cost and QALY of our opt-out intrapartum universal rapid HIV screening strategy with the two current alternative standards of care:* standard of care I*, opt-out rapid HIV screening limited to patients without previous third-trimester HIV screening;* standard of care II*, opt-out rapid HIV screening limited to patients without any prenatal HIV screening.

After Institutional Review Board approval from the MedStar Health Research Institute (#2015-069), we developed a decision-tree model to analyze the cost-effectiveness of universal, opt-out, rapid HIV screening of pregnant patients admitted for delivery (spontaneous labor or scheduled induction of labor or cesarean delivery), taking into account the incremental lifetime costs and the loss of QALY associated with MTCT and PAH. We designed our model using the estimated values of parameters listed in Table 2. In our decision-tree model, we took into account the different potential combinations of prenatal HIV screening algorithms, adherence to screening algorithms, and screening results. We used the incidence and prevalence of reproductive aged women HIV in DC to derive the probability maternal HIV acquisition and seroconversion between prenatal HIV screening and intrapartum HIV screening. Taking into consideration the spectrum of clinical presentations to labor and delivery, we then applied the different intrapartum screening strategies to potential clinical scenarios (universal rapid HIV screening, standard of care I, and standard of care II) (Table 1). Using the estimated maternal intrapartum rapid HIV results/maternal intrapartum HIV status, based on the intrapartum screening strategies, we projected the different risks of MTCT for each strategy and accordingly the risk of a HIV exposed versus a HIV infected neonate. From our model's findings, for each screening strategy, we then calculated the estimated additional cost and reduced QALYs for an infected neonate.

In our model, we considered the incidence of HIV during pregnancy and calculated the probability that women with a negative prenatal screening at T1 acquire HIV by the time of delivery. We also took into account the fact that only universal rapid HIV screening would identify new cases of maternal HIV acquired between the third trimester and delivery, allowing for the possibility of maternal AZT prophylaxis during delivery, CD (if indicated), immediate neonatal antiretroviral prophylaxis, diagnostic testing, counseling against breastfeeding, and neonatal ART as indicated. We further took into account the sensitivity and specificity of the prenatal HIV screening and rapid HIV test and the possibility of incomplete maternal prophylaxis secondary to rapid labor or other time constraints. Given the lack of evidence that CD is protective against MTCT in labor or after ROM, we did not include a protective effect of CD in these clinical circumstances in our model. Upon review of the published literature, however, we noted a higher proportion of CD rate among these pregnancies with positive rapid HIV tests, likely secondary to providers' concerns for prevention of MTCT above and beyond the guidelines. To estimate the proportion of CD among pregnancies with a positive rapid HIV test in our model, we used the proportion of CD found by Bulterys et al. to reflect clinical practice, regardless of the unknown protective effect [14].

**Figure 1:**
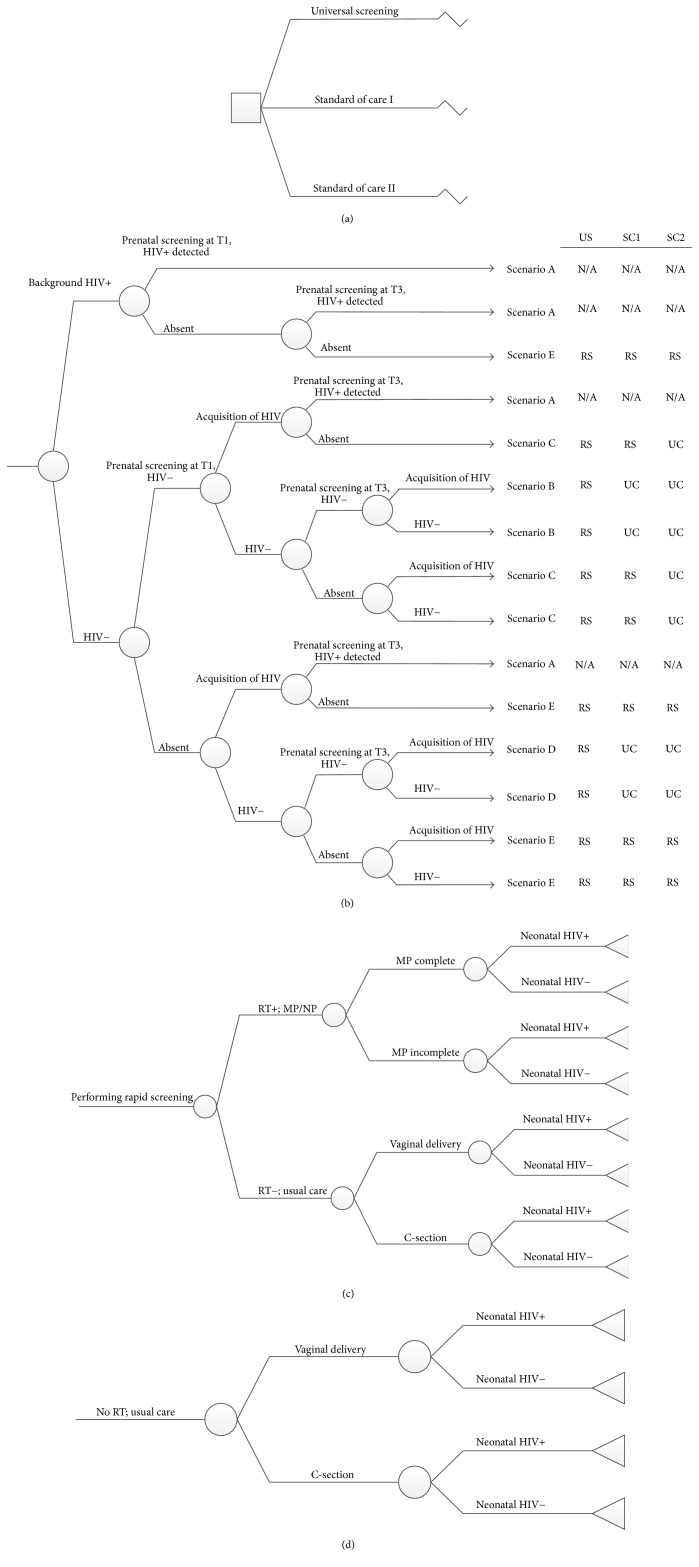
Simplified depiction of decision-tree used for the analysis. (a) Three strategies, universal rapid HIV screening, standard of care I, and standard of care II, are compared in the analysis. (b) Scenarios based on prenatal HIV screening and the screening result. (For simplicity, the figure assumes 100% sensitivity and 100% specificity for the prenatal HIV screening.) (c) The care pattern for patients undergoing intrapartum rapid screening. (d) The care pattern for patients not undergoing intrapartum rapid screening. US: universal screening; SC1, standard of care I; SC2, standard of care II; RS, rapid screen; UC, usual care; MP, maternal prophylaxis; NP, neonatal prophylaxis; T1, first trimester; T3, third trimester.

Regardless of the HIV screening strategy, patients with known HIV would have been treated per standard of care (with maternal ART, maternal prophylaxis and CD as indicated, and neonatal prophylaxis) and therefore would not undergo intrapartum universal rapid HIV screening and would have no impact on the incremental cost and effectiveness of our analysis.  Figure 1 is a simplified depiction of decision-tree used for the analysis.

### 2.2. Parameters

The measures of costs included the materials and services of the rapid HIV test, associated treatments for patients who tested positive, additional costs of CD, and the lifetime medical costs for a newborn with PAH. We assumed that other costs were the same across the three strategies and were not included in the cost analysis. We adjusted all monetary values to the 2015 USD. We applied a 3% discount rate to estimate the savings in both QALY and lifetime medical costs in present values by preventing one case of PAH. Detailed values for each input parameter are listed in Table 2. We obtained these values and associated assumptions from a combination of previously published peer reviewed medical literature and statistics from the Washington DC Department of Health.

### 2.3. Cost-Effectiveness Analysis

We used the incremental cost-effectiveness ratio (ICER), defined as the incremental cost divided by incremental health gains, for the cost-effectiveness analysis. We estimated the incremental cost per QALY of universal rapid HIV screening, using standard of care I and standard of care II as the references, respectively. $50,000 to $100,000 per QALY is considered the standard acceptable range for a cost-effective intervention [22, 23]; however, Ubel and colleagues and others argue that this range is overly conservative and that even the upper threshold of $100,000 is still a too low value to put on life [24]. In our study, we used the upper limit of the conservative estimate, $100,000, as the threshold for a cost-effective intervention.

### 2.4. Sensitivity Analysis

For further comparison between the proposed strategy (universal rapid HIV screening) and both the standard of care in the DC area (standard of care I) and standard of care II, we conducted univariate sensitivity analyses to estimate the impact of selected variables on the model, including HIV prevalence and cumulative incidence, QALY saved by preventing one case of MTCT, and lifetime medical cost of treatment for a neonate with PAH. The range for each parameter incorporates the highest and lowest values from the medical literature or expert opinions. Considering the argument that a higher lifetime medical cost in treating HIV leads to a lower level of difference in QALY, compared to a HIV negative individual, we also conducted a two-way sensitivity analysis by varying the cost and the difference in QALY.  Table 3 shows the range of variation of parameters for these variables.

## 3. Results

Among the three strategies, the universal screening appeared to have the highest estimated cost and QALYs per 10,000 pregnant women (Table 4). Using standard of care I as the reference strategy, the incremental cost of the universal screening per 10,000 patients was $39,827.83 with 5.01 QALYs saved. The ICER was estimated to be $7,943.45. Using standard of care II as the reference, the ICER for the universal screening was $3,610.58. Both estimates are less than $100,000—a conservative threshold for a cost-effective intervention. Standard of care I achieved higher QALYs than standard of care II with lower estimated cost, indicating that standard of care II is inferior.

Table 5 shows the results of the sensitivity analyses for the ICER of universal rapid HIV screening to standard of care I. For the QALY saved, lifetime medical costs, rapid test cost, cost of neonatal prophylaxis, and HIV prevalence, the calculated ICERs were below the threshold for the extreme values of range, indicating that the results remained robust. We obtained similar results in second sensitivity analyses comparing universal rapid HIV screening to standard of care II (Table 6).

**Figure 2:**
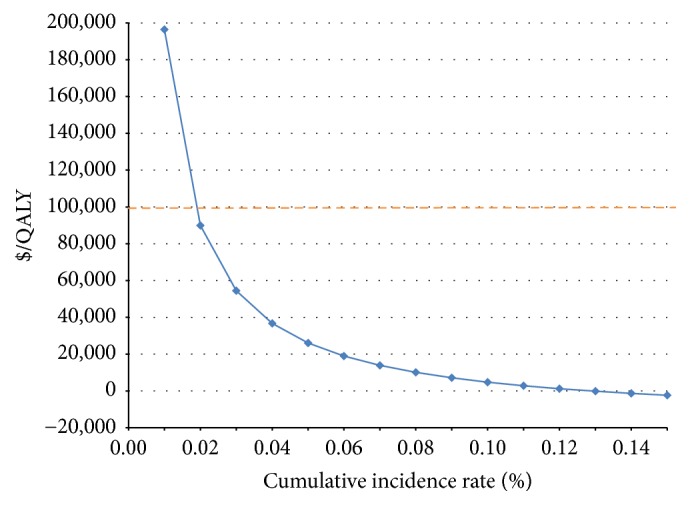
One-way sensitivity analysis: impact of annual cumulative incidence on ICER (universal screening relative to standard of care in the DC area).

**Figure 3:**
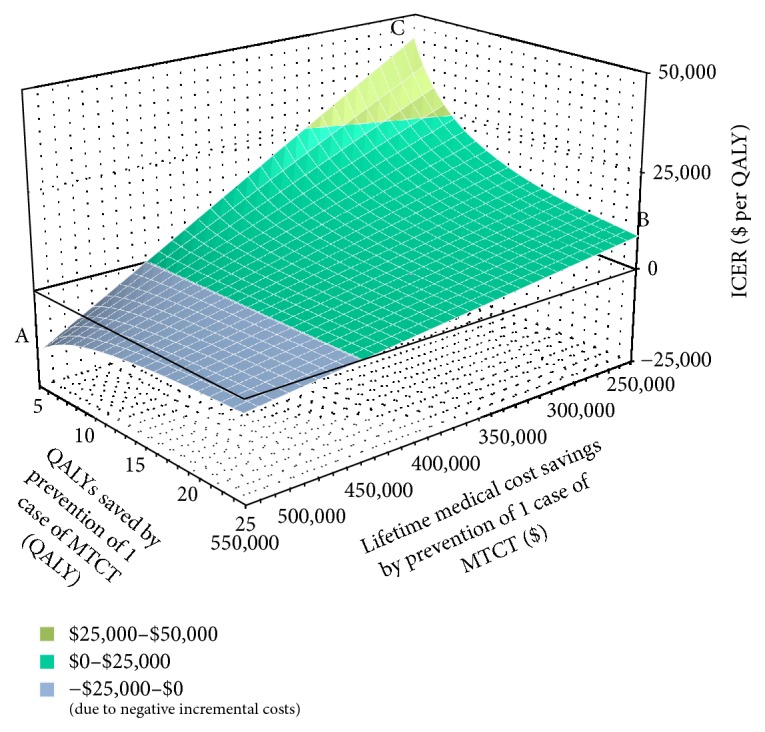
Two-way sensitivity analysis: impact of QALY saved per prevented case of MTCT and lifetime medical costs of PAH on ICER (universal screening relative to standard of care in the DC area).

Figures 2 and 3 illustrate the sensitivity analyses for the ICER of universal screening relative to standard of care in the DC area. As demonstrated in Figure 2, favorable ICER was dependent upon a high cumulative incidence of HIV. Specifically, if the annual cumulative incidence rate were less than 0.02% (i.e., less than a fifth of the rate in DC) the incremental cost-effectiveness ratio for the expanded program would exceed $89,926.94/QALY, approaching the cost-effectiveness threshold ($100,000/QALY). When the incidence rate approaches 0.13% (i.e., about 1.5 times in DC), the universal screening is both less expensive and more effective screening strategy.

Figure 3 shows the combined impact of the QALYs saved from averting one case of MTCT and the lifetime medical cost of PAH on the estimate of ICER, based on a two-way sensitivity analysis. In the direction of inverse relationship between the QALYs saved and the lifetime medical cost (A↔B), the scenario with low cost and large QALYs saved (point B) has higher ICER compared to the one with high cost and small QALYs saved (point A). However, the universal rapid HIV testing still demonstrated a cost-effective option ($8,762.90/QALY; point B). The analysis further indicated that low lifetime medical cost and low incremental QALY lead to high ICER. However, even using the highest estimate of $250k lifetime medical costs and the lowest level of QALY averted (5), the ICER remained below the $100,000 threshold ($43,814.50/QALY; point C).

## 4. Discussion

Diagnosis and treatment of maternal HIV is crucial to eliminating MTCT and the eventual eradication of HIV. In our model, we considered the costs of the rapid HIV test and of the preventive interventions, taking into account sensitivity and specificity of the rapid HIV test at time of delivery and of previous prenatal screening, along with the probability of having had previous prenatal screening.

Our sensitivity analysis compared universal rapid HIV screening to “standard of care I,” and as we hypothesized we found universal rapid HIV screening to be more cost-effective in areas with an annual cumulative incidence of HIV in women of reproductive age greater than 0.02%. In areas with a very low cumulative incidence of HIV, specifically less than 0.02%, universal rapid HIV screening will be of questionable cost-effectiveness. Using the threshold of $100,000 for a cost-effective intervention, “standard of care I” was only more favorable than universal rapid HIV screening or “standard of care II” if the annual cumulative incidence was less than 0.01% (where ICER_universal  rapid  HIV  screening  to  “standard  of  care  I”_ = $196k/QALY; ICER_“standard  of  care  I”  to  “standard  of  care  II”_ = $82k/QALY). To expand our model, we varied the values of HIV prevalence and incidence to assess whether the universal rapid HIV screening would still be the superior strategy in different communities. We found that HIV prevalence was not the key driver in our sensitivity analysis, but rather cumulative incidence. The cumulative incidence highly affected the estimated ICER; specifically lower cumulative incidence resulted in higher ICER. The decreased impact of HIV prevalence in our sensitivity analysis was secondary to the high sensitivity of prenatal HIV screening (100% in our assumption); pregnant women with known HIV would be identified in the prenatal period and treated per guidelines, as discussed previously, leading to a limited impact in our model.

This model can be applied to other communities to determine whether intrapartum universal rapid HIV screening is cost-effective for their patient population, depending upon their local HIV incidence and the costs of interventions to test and prevent MTCT. Within the United States, universal rapid HIV screening would likely be cost-effective and should be considered in other high incidence areas such as Baltimore, Miami, New Orleans, and New York City. By the substituting local costs (such as the cost of maternal AZT) and local probabilities (such as maternal cumulative incidence in the community and CD rate), communities can individualize this model to gauge the cost-effectiveness of universal rapid HIV screening at time of delivery. We are currently developing a computer and mobile device based application to allow adaptation of the model to other communities.

The model presented in this paper is a simplified representation of a complicated medical and psychosocial scenario. For example, in patients who arrive in active labor or after rupture of membranes, there is limited literature on the benefit of CD and we assumed no protective effect in our model. Our model used the published experience of other centers to provide the most accurate real-world model we could create. Our model focuses on the health outcomes of HIV exposed neonates at high risk of MTCT and the economic burden of their medical care. We chose QALY as the basis for our model, which takes into account both health-related quality of life and adjusted life-expectancy [24]. Any model is limited in its ability to take into account all of the medical, economic, and psychosocial components at play in real life. We reviewed and considered multiple additional outcome measures that were not ultimately included in our model, such as maternal benefits and burdens related to universal rapid HIV screening at time of delivery, the lifetime nonmedical costs (such as loss of productivity) or the other opportunity costs affecting social activities due to the HIV, potential benefits to the health care institutions that adopt universal rapid HIV screening, such as potential savings from an avoided lawsuit, and other potential reduction of societal burdens, including risk of transmission to other individuals by a PAH patient during lifetime. While we recognize the inherent simplification and limitations of our model, we have incorporated the major driving medical and economic factors in our analysis.

Improvement in medical technology has facilitated better treatment and outcomes for individuals living with HIV, resulting in longer life-expectancy with better quality of life. The gap between the levels of QALY for a HIV infected neonate and an uninfected neonate has been decreasing and, by living longer, the lifetime medical cost of treating PAH is inevitably increasing. Even with the most extreme scenario using the lowest QALYs saved and the highest lifetime medical cost obtained from our references for the two-way sensitivity analysis, the ICER of universal rapid HIV screening to standard of care I is still below the $100,000/QALY threshold, indicating the impact of the potential adverse association between the QALYs saved and the lifetime medical cost on the decision is limited. That said, we acknowledge the possible underestimate of ICERs in our study.

Our model does not involve complicative logic, such as repeated cycles, event tracking, or stage transition. Although we considered both static decision-tree and microsimulation modeling, we opted for the decision-tree, as we did not find that microsimulation conferred a more in-depth understanding of cost-effectiveness of universal intrapartum HIV screening.

## 5. Conclusion

We developed a novel model to test the cost-effectiveness of HIV screening protocols in pregnancy and those intrapartum. Through our model, we justify opt-out universal rapid HIV screening at delivery to be a highly cost-effective strategy for preventing MTCT in high incidence areas.

## Figures and Tables

**Table 1:** Explanation of three strategies of care at delivery based on scenarios at prenatal care.

Scenario	Universal screening	Standard of care I	Standard of care II
(A) HIV+ (identified during or before prenatal HIV screening)	Treated per standard care for known HIV, no intrapartum rapid HIV screening		
(B) T1, T3 negative prenatal HIV screening with negative result	Intrapartum rapid HIV screening	Usual care	Usual care
(C) T1 negative prenatal HIV screening (no T3 screening)	Intrapartum rapid HIV screening	Performing rapid screening	Usual care
(D) T3 negative prenatal HIV screening with negative result (no T1 screening)	Intrapartum rapid HIV screening	Usual care	Usual care
(E) No T1 and T3 prenatal HIV screening	Intrapartum rapid HIV screening	Intrapartum rapid HIV screening	Intrapartum rapid HIV screening

T1, the first trimester; T3, the third trimester.

**Table 2:** Input parameters.

Variable	Value	Reference
*Probability variables*		
Prevalence of HIV at initial test	1.9%	[17]^†^
Cumulative incidence of HIV/year^*α*^	0.087%	[17]^†^
Sensitivity of prenatal HIV test	100.0%	[18]
Specificity of prenatal HIV test	99.9%	[18]
Initial HIV test compliance	90.4%	[19]
3rd-trimester HIV test compliance	80.0%	[19]
Sensitivity of rapid test	99.7%	Package insert^‡^
Specificity of rapid test	99.9%	Package insert^‡^
Cesarean delivery rate	32.7%	MWHC, DC^*₸*^
Probability of vertical transmission for a vaginal delivery (without maternal prophylaxis (MP) or neonatal prophylaxis (NP))	25.5%	[12]
Probability of vertical transmission for a cesarean delivery before active labor or rupture of membranes (without maternal prophylaxis or neonatal prophylaxis)	10.4%	[10]
Probability of vertical transmission with maternal and neonatal prophylaxis	2.8%	[20]
Probability of vertical transmission with only neonatal prophylaxis	5.7%	[20]
*Cost variables *		
Cost of rapid test	$14.98	Medicare Clinical Diagnostic Laboratory Fee Schedule^*∗*^
Cost of maternal prophylaxis (with zidovudine (AZT))	$61.30	Calculated^∫ ^
Cost of neonatal prophylaxis (AZT + Nevirapine (NVP) per US guidelines)	$185.00	Calculated^*₸*^
Cost of usual care and cesarean delivery	$9,417.60	Medicare physician fee schedule and HCUPnet^*ϵ*^
Cost of usual care and vaginal delivery	$6,473.24	Medicare physician fee schedule and HCUPnet^*ϵ*^
Lifetime additional medical cost for PAH in present value	$318,147.00^¶⌉^	[21]
*Other variables*		
QALY saved if one case of MTCT was prevented in present value	19^⌉^	[21]

^†^Data were based on women between ages of 14 and 45 at the end of 2012, obtained/derived from Department of Health, Government of the District of Columbia. ^*₸*^Data were provided by the Women's and Infants' Services Department of MedStar Washington Hospital Center in 2015. ^‡^Clearview HIV 1/2 STAT-PAK package insert. ^∫ ^Assuming adequate treatment of 2 mg/kg loading dose, + 1 mg/kg/hr times 3 hrs prior to delivery, and ideal body weight of a 64-inch female + 25 lb weight gain during pregnancy = 155 lb or 70 kg. Published cost of AZT is 35.03 for 200 mg (20 mL of 10 mg/mL). Cost of AZT based on 70 kg woman for adequate prophylaxis: (140 mg loading dose + 70 mg/hr × 3 hrs = 350 mg) = 350 mg × $35.03/200 mg = $61.30. ^*∗*^Cost data obtained from the Fisher Scientific Website https://www.fishersci.com/us/en/catalog/search/products?keyword=4th+generation+rapid+hiv+test&nav.^¶^Updated to 2015 dollars. ^⌉^The value was derived from 28 minus 9 from the reference. A 3% discount rate has been applied to indicate a present value. ^*α*^A cumulative incidence, or incidence proportion, is the proportion of a initially disease-free population that developed disease during a specified period of time, http://www.cdc.gov/ophss/csels/dsepd/ss1978/lesson3/section2.html. With the annual cumulative incidence available, we derived 12 weeks, 14 weeks, and 26 weeks of cumulative incidences needed in our model by assuming no temporal trend of the risk. ^*ϵ*^Costs of vaginal delivery and Cesarean delivery were the combination of hospital costs and physician costs. The hospital costs were obtained based on the DRG codes and the division, using HCUPnet online tool http://hcupnet.ahrq.gov/. Costs of physician services were based on the Medicare physician fee schedule (https://www.cms.gov/medicare/medicare-fee-for-service-payment/physicianfeesched/) with associated HCPCS codes and the location.

**Table 3:** Sensitivity analysis values and ranges.

Variable	Range	Reference
QALY saved per prevention of 1 case of MTCT	5–25^⌉^	[20, 25–30]
Estimated lifetime medical cost of PAH	$250k–$550k^¶⌉^	[20, 25–28, 31, 32]
HIV prevalence	0.5%–10%	‡
HIV annual cumulative incidence	0.01%–0.15%	‡
Cost of rapid test	$11.90–$16.32	*⊣*
Neonatal prophylaxis	$185–$210	€

^¶^Range was set to cover the cost value obtained from the references (after it was updated to 2015 dollars).  ^⌉^A 3% discount rate was applied to indicate a present value. ^‡^The range was set to examine the impact of extreme value on the estimated outcomes, not necessarily indicating a highest or lowest rate in District of Columbia. ^⊣^Range was set based on the minimum and maximum of Medicare Clinical Diagnostic Laboratory Fee Schedule among different locations for the same service. ^€^We also considered three-drug prophylaxis, with Lamivudine in addition to AZT and Nevirapine. 240 ml of the 10 mg/ml oral solution costs $76.52, per the Neonatal Pharmacy Department of MedStar Washington Hospital Center. By the US AIDS info guidelines, a 3500 g neonate requires 784 mg total of Lamivudine ($25), in addition to AZT and Nevirapine ($185).

**Table 4:** Estimated cost and effectiveness per 10,000 pregnant women.

Strategy	Estimated cost ($)	Estimated QALYs	Reference: standard of care I			Reference: standard of care II		
			Incremental cost ($)	Incremental QALY	ICER ($/QALY)	Incremental cost ($)	Incremental QALY	ICER ($/QALY)
Universal rapid HIV screening	75,606,835.51	279,941.73	39,827.83	5.01	7,943.45	26,968.76	7.47	3,610.58
Standard of care I	75,567,007.68	279,936.71	- -	- -	- -	- -	- -	- -
Standard of care II	75,579,866.75	279,934.26	12,859.07	−2.46	−5,236.97	- -	- -	- -

**Table 5:** Univariate sensitivity analysis of universal rapid HIV screening relative to standard of care I.

Variable	Detected range	Estimated incremental cost ($)	Estimated incremental QALY	Incremental cost-effectiveness ratio ($/QALY)
QALY per prevention of 1 case of MTCT	5~25	39,827.83	1.32~6.60	30,185.10~6,037.02
Estimated lifetime medical cost of PAH	$250k~$550k	57,811.19~(−21,356.02)	5.01	11,530.13~(−4,259.34)
HIV prevalence	0.5%~10%	40,396.22~36,539.30	5.09~4.60	7,943.45~7,943.45
HIV annual cumulative incidence	0.01%~0.15%	113,233.84~(−20,316.76)	0.58~8.65	196,399.01~(−2,348.85)
Cost of rapid test	$11.90~$16.32	15,661.65~50,341.69	5.01	3,123.63~10,040.38
Cost of neonatal prophylaxis	$185~$210	39,827.83~40,063.20	5.01	7,943.45~7,990.39

**Table 6:** Univariate sensitivity analysis of universal rapid HIV screening relative to standard of care II.

Variable	Detected range	Estimated incremental cost ($)	Estimated incremental QALY (10^−5^)	Incremental cost-effectiveness ratio ($/QALY)
QALY saved per prevention of 1 case of MTCT	5~25	26,968.76	1.97~9.83	13,720.22~2,744.04
Estimated lifetime medical cost of PAH	$250k~550k	53,759.00~(−64,178.29)	7.47	7,197.27~(−8,592.20)
HIV prevalence	0.5%~10%	27,353.64~24,741.98	7.58~6.85	3,610.58~3,610.58
HIV annual cumulative incidence	0.01%~0.15%	136,311.47~(−62,625.81)	0.86~12.89	158,710.06~(−4,860.01)
Cost of rapid test	$11.90~$16.32	(−2,660.25)~39,859.31	7.47	(−356.16)~5,336.37
Cost of neonatal prophylaxis	$185~$210	26,968.76~27,267.68	7.47	3,610.58~3,650.60

## Additional Points

*Condensation*. Universal rapid HIV screening at time of delivery to decrease mother-to-child transmission appears cost-effective in populations with high HIV incidence in the United States.
